# P2X4 Receptor Function in the Nervous System and Current Breakthroughs in Pharmacology

**DOI:** 10.3389/fphar.2017.00291

**Published:** 2017-05-23

**Authors:** Leanne Stokes, Janice A. Layhadi, Lucka Bibic, Kshitija Dhuna, Samuel J. Fountain

**Affiliations:** ^1^School of Pharmacy, University of East Anglia, Norwich Research ParkNorwich, United Kingdom; ^2^School of Biomedical and Health Sciences, RMIT University, BundooraVIC, Australia; ^3^Biomedical Research Centre, School of Biological Sciences, University of East AngliaNorwich, United Kingdom

**Keywords:** ATP, P2X4, microglia, pain, antagonist, ivermectin

## Abstract

Adenosine 5′-triphosphate is a well-known extracellular signaling molecule and neurotransmitter known to activate purinergic P2X receptors. Information has been elucidated about the structure and gating of P2X channels following the determination of the crystal structure of P2X4 (zebrafish), however, there is still much to discover regarding the role of this receptor in the central nervous system (CNS). In this review we provide an overview of what is known about P2X4 expression in the CNS and discuss evidence for pathophysiological roles in neuroinflammation and neuropathic pain. Recent advances in the development of pharmacological tools including selective antagonists (5-BDBD, PSB-12062, BX430) and positive modulators (ivermectin, avermectins, divalent cations) of P2X4 will be discussed.

## Introduction

Purinergic P2X receptors are involved in mediating a multitude of functions in health and disease via the actions of extracellular adenosine 5′-triphosphate (ATP) (North, 2002; Burnstock and Kennedy, 2011; Khakh and North, 2012). Although ATP has long been recognized as an intracellular energy source, its acceptance as an extracellular signaling molecule has taken considerably longer (Burnstock, 2006). It is now widely recognized that extracellular ATP acts as either sole neurotransmitter or crucial co-neurotransmitter in most nerves in both the peripheral nervous system and central nervous system (CNS) (Burnstock, 2006; Khakh and North, 2012). For example, under physiological conditions, astrocytes and neurons are responsible for releasing small amounts of ATP, which can then act on purinergic receptors – either P2X ion channels or P2Y G protein-coupled receptors - expressed on both neurons and glial cells. In healthy tissues, ATP released from cells is tightly regulated with cell surface ectonucleotidases serving to terminate purinergic signaling (Cardoso et al., 2015) (this is analogous to the activity of cholinesterases at cholinergic synapses). However, when released from injured cells, ATP can initiate inflammation and further amplify and sustain cell-mediated immunity through P2 receptors (Idzko et al., 2014).

P2X receptors are non-selective cation channels that open in response to ATP binding, allowing the rapid flow of ions (K^+^, Na^+^, Ca^2+^) across the membrane. Seven genes encode P2X receptor subunits (P2X1-7) that are expressed in all tissues in humans and mice (North, 2002; Khakh and North, 2006). P2X receptors share a common topology assembling as trimers (Hattori and Gouaux, 2012), with evidence of both homomeric and heteromeric assembly (Saul et al., 2013). Different subtypes are involved in specialized functions depending on their distribution and biophysical properties (Surprenant and North, 2009). These differences provide an opportunity for tissue-specific inhibition of one subtype without affecting the function of others. Such challenges represent a pharmacological opportunity to intervene in physiological processes including modulation of synaptic transmission, taste, pain sensation, and inflammation (Khakh and North, 2006).

Progress on several fronts has revealed the involvement of P2X4 in a variety of pathophysiological processes. For example, P2X4 are implicated in neuropathic pain (Tsuda et al., 2003, 2009a; Coull et al., 2005; Ulmann et al., 2008), inflammatory pain (Ulmann et al., 2010), epilepsy (Ulmann et al., 2013), lung surfactant secretion (Miklavc et al., 2013), alcohol intake and preference (Wyatt et al., 2014; Franklin et al., 2015), morphine-induced hyperalgesia (Ferrini et al., 2013), cardiac function (Yang et al., 2014) and P2X4 deficiency is associated with sociocommunicative impairments (Wyatt et al., 2013) and altered flow-dependent blood vessel remodeling (Yamamoto et al., 2006). However, the study of P2X4 has been seriously hampered by an absence of selective pharmacological tools. While the discovery of selective antagonist molecules against P2X4 would facilitate the further elucidation of their physiological roles, their application might also hold promises to lead us into novel therapeutics for human diseases. Here we aim to present a broad, comprehensive overview of P2X4 receptors, their function in the CNS, and their emerging pharmacology.

## P2X4; Ion Channel Structure and Permeability

The P2X4 receptor, now simply termed P2X4 using the latest IUPHAR nomenclature (Di Virgilio et al., 2015), is a ligand-gated ion channel belonging to the P2X receptor family. This family of ion channels are activated by extracellular ATP and function as non-selective cation channels permitting Na^+^, K^+^ and Ca^2+^ ion fluxes (North, 2002). The Ca^2+^ permeability of P2X4 is the highest among the P2X family (Egan and Khakh, 2004). Measurement of the fractional calcium current (Pf%) through P2X4 using the patch clamp photometry technique (measuring the ATP-induced inward current and the concomitant decrease in the emission of fura-2 fluorescence excited at 380nm) yields values of 11% and 15% for rat P2X4 and human P2X4, respectively (Egan and Khakh, 2004).

P2X subunits are arranged into trimeric structures in the plasma membrane, either in homomeric or heteromeric formations (Saul et al., 2013). This trimeric organization of P2X4 was confirmed in 2009 by solving the crystal structure of a zebrafish P2X4 (zP2X4) in the closed state at 3.1A resolution (Kawate et al., 2009). Three inter-subunit binding sites for ATP were defined in the P2X extracellular domain. In 2012 a crystal structure for the ATP-bound state of zP2X4 was solved revealing the open pore conformation of the channel (Hattori and Gouaux, 2012). This also gave structural insight into the mechanics of channel opening in response to ATP binding; fenestrations open up in close proximity to the plasma membrane providing lateral access pathways for cation entry to the ion channel pore (Samways et al., 2011; Hattori and Gouaux, 2012; Gao et al., 2015). This movement of the extracellular domains upon agonist binding leads to iris-like rotational movements of the transmembrane domains lining the channel pore thus describing the predicted activation mechanism of P2X4 channels (Hattori and Gouaux, 2012). Fast-scanning atomic force microscopy has also been used to reveal the trimeric structure of P2X4 and movement of subunits following ATP binding (Shinozaki et al., 2009).

The P2X4 subtype can heteromerically assemble with other P2X members including P2X6, P2X7 and P2X1 (Le et al., 1998; Nicke et al., 2005; Antonio et al., 2011). These studies have typically used immunoprecipitation of P2X4-containing protein complexes and detection of other P2X using antibodies in combination with altered functional and pharmacological properties as major pieces of experimental evidence. For example, Xenopus oocytes expressing both P2X1 and P2X4 exhibit a slowly desensitizing current similar to homomeric P2X4 but in the presence of P2X1 there is significant activation by αβ-MeATP and inhibition by suramin (Nicke et al., 2005), properties not seen with either P2X4 or P2X1 alone. The reported interaction with P2X7 is much more contentious. Evidence first suggested that P2X4 and P2X7 could form heterotrimeric complexes (Boumechache et al., 2009) but then further investigations revealed that homotrimers of P2X4 and P2X7 may interact with each other (Antonio et al., 2011).

## Permeability

Khakh and colleagues first described different permeability states for P2X4, denoting two states (I_1_ and I_2_ state) in response to a sustained exposure to ATP (Khakh et al., 1999a). This secondary permeability state (I_2_) displays increased NMDG^+^ permeation suggestive of a larger channel pore size and a dynamic change in ion selectivity. This concept has recently been challenged and V_rev_ changes recorded during NMDG^+^/Na^+^ bi-ionic conditions is thought to be due to alterations in intracellular ion depletion and accumulation (Li et al., 2015). This data can be interpreted as lack of evidence for dynamic changes in ion conductance but whether P2X channels are directly permeable to large cations such as NMDG^+^ still warrants further investigation. Further evidence for a secondary permeability state (or permeation of large organic cations) for P2X4 comes from the use of membrane impermeant fluorescent dyes such as YOPRO-1 iodide and ethidium bromide (Khakh et al., 1999a; Bernier et al., 2012). Whether these dyes permeate directly through the P2X4 channel or an associated conduit is not yet clear. Much evidence exists for a large secondary permeation pathway activated by P2X7 in numerous cell types. Classically this is measured by the use of dye uptake assays (in particular such as ethidium bromide, YOPRO-1 iodide, Lucifer yellow). Controversy surrounds the underlying mechanism of action for P2X7 with some studies suggesting a second protein may carry the dyes, such as pannexin-1 (Pelegrin and Surprenant, 2006) or anoctamin-6 (Ousingsawat et al., 2015) and others suggesting that P2X7 itself can allow passage of large molecules (Browne et al., 2013). The physiological role of this permeability pathway for large cations is still unclear for P2X7 and P2X4 channels, and indeed those other ligand-gated ion channels demonstrating a similar phenomenon (Chung et al., 2008; Banke et al., 2010).

## Expression of P2X4 in the CNS

P2X4 was originally cloned from rat brain cDNA in 1996 (Soto et al., 1996a) and was the first P2X receptor detected in CNS neurons and blood vessels. P2X4 was subsequently cloned from a human brain sample (Garcia-Guzman et al., 1997) and was also found to exhibit a broad tissue expression pattern. Amongst the P2X receptor subtypes, P2X4, along with P2X2 and P2X6, has been shown to be the most widespread and abundantly expressed functional ATP-gated purinergic receptor in the CNS, being found in most neurons and glial cells (Buell et al., 1996; Soto et al., 1996a,b; Tsuda et al., 2003; Guo et al., 2005; Amadio et al., 2007; Vazquez-Villoldo et al., 2014). This has been illustrated at both mRNA and protein level by *in situ* hybridisation, immunohistochemistry, PCR and immunoblotting (Soto et al., 1996b; Lê et al., 1998; Bo et al., 2003).

## Neurons

Throughout the CNS, expression of P2X4 is widely observed in neurons. In the original paper cloning P2X4 from rat brain, Soto and colleagues demonstrated high levels of P2X4 mRNA in rat dentate gyrus granule cells, CA1/CA3 pyramidal cells, cerebellar cortex Purkinje cells, and neurons of the pontine nucleus (Soto et al., 1996a). Electron microscopy analysis suggests P2X4 localisation in peri-synaptic regions of post-synaptic terminals and on pre-synaptic terminals (Rubio and Soto, 2001). Immunohistochemistry also shows P2X4 to be expressed in GABAergic interneurons and GABAergic spiny neurons of the rat striatum and substantia nigra (Amadio et al., 2007).

The hypothalamus and anterior pituitary gland abundantly express P2X4 and this receptor may be involved in regulation of hypothalamo-pituitary functions in the CNS, as reviewed in Stojilkovic (2009). Immunohistochemistry studies identified P2X4 on paraventricular nucleus neurons projecting to the rostral ventrolateral medulla and a potential role in regulating sympathetic nerve activity (Cham et al., 2006). Paraventricular neurons, arcuate nucleus GnRH neurons and secretory cells of the anterior pituitary all express P2X4 as illustrated through molecular biology techniques in combination with electrophysiology (Zemkova et al., 2010). Functional P2X4 receptors have also been identified in lactotrophs (He et al., 2003), and in the posterior pituitary system functional P2X4 responses have been recorded from supraoptic neurons (Vavra et al., 2011; Stojilkovic and Zemkova, 2013). P2X4 has also been demonstrated to be expressed in somatosensory cortical neurons (Lalo et al., 2007), nodose ganglion neurons (Tan et al., 2009), trigeminal neurons (Luo et al., 2006), vestibular ganglion neurons (Ito et al., 2010), retinal ganglion and bipolar cells (Wheeler-Schilling et al., 2001) and in spinal cord neurons (Bardoni et al., 1997; Kobayashi et al., 2005).

P2X4 has been implicated in physiological functions in the CNS including modulation of neurotransmission and synaptic strengthening (Rubio and Soto, 2001; Sim et al., 2006; Baxter et al., 2011). In the hippocampus P2X4 expression on pyramidal neurons is thought to contribute to synaptic plasticity and long-term potentiation (LTP). One of the initial studies that elaborated a role for P2X4 in LTP was performed in mice with a global deficiency in the *p2rx4* gene (P2X4^-/-^) (Sim et al., 2006). Extracellular recording of field potentials from the CA1 region of the hippocampus in these P2X4-deficient mice revealed reduced synaptic facilitation and induction of LTP compared to wild-type counterparts. In addition, ivermectin, a positive allosteric modulator of P2X4, increased LTP in wild-type mice but was ineffective in the P2X4^-/-^ mice (Sim et al., 2006). This suggested that P2X4 contributes to strengthened synaptic activity during LTP and it is hypothesized that calcium entry through sub-synaptic P2X4 contributes to synaptic strengthening by NMDA receptor incorporation (Baxter et al., 2011).

Studies have also investigated cross-talk between P2X4 and other ion channels in neurons, in particular GABA(A) receptors (Jo et al., 2011) and nicotinic acetylcholine receptors (Khakh et al., 2000). In hypothalamic neurons increased expression of P2X4 is associated with a reduction in GABAergic currents (Jo et al., 2011). There is some evidence for a physical coupling between P2X4 and GABA(A) receptors and this may play a role in regulating synaptic signaling (Jo et al., 2011). A similar cross-talk has been demonstrated for P2X2 receptors and GABA(A) receptors and this cross-talk appears to be a general mechanism for the regulation of GABAergic signaling, as reviewed in Shrivastava et al. (2011). Therefore, P2X4 may be involved in regulating excitatory vs. inhibitory neurotransmission in neurons, acting as a neuromodulator. The contribution of P2X4 in this process will likely become clearer in the future with the development of selective pharmacological tools and more knockout mouse studies.

## Glial Cells

In the CNS P2X4 plays a role in modulating synaptic transmission and communication between neurons and neighboring glial cells. Glia are the most abundant cell type accounting for >70% of total cells in the CNS and can be classified into three main types; astrocytes, oligodendrocytes and microglia.

The role of P2X4 in microglial cells has received much attention in the last decade. Microglial cells, known as the resident macrophages in the CNS, originate from the yolk sac and are related to myeloid immune cells (Kettenmann et al., 2011; Saijo and Glass, 2011). Immunohistochemistry analysis has revealed abundant P2X4 reactivity on microglia within the brain and spinal cord (Tsuda et al., 2003; Ulmann et al., 2008). Despite the fact that P2X4 is abundantly expressed in microglial cells, the majority of labeled P2X4 appears to be predominantly localized to intracellular lysosomal compartments (Qureshi et al., 2007; Toyomitsu et al., 2012). P2X4 has been suggested to play a role in regulating the activation and migration of microglial cells at sites of injury (Guo et al., 2005; Schwab et al., 2005). Following peripheral nerve injury, the expression of P2X4 is up-regulated in activated spinal cord microglia at the transcriptional, translational and post-translational levels (Ulmann et al., 2008). Several factors that have been associated with regulating P2X4 expression include the chemokine CCL21 (Biber et al., 2011), fibronectin (Nasu-Tada et al., 2006; Tsuda et al., 2008), and the cytokine interferon gamma (IFN-γ) (Tsuda et al., 2009b), while activation of the chemokine receptor CCR2 is a key factor involved in post-translational regulation of P2X4 (Toyomitsu et al., 2012). Fibronectin and IFN-γ can regulate P2X4 expression through the IRF5 transcription factor in microglia (Masuda et al., 2014).

**FIGURE 1:**
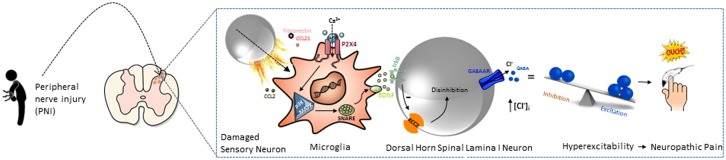
**Postulated role of P2X4 signaling in chronic pain.** Peripheral nerve injury (PNI) activates microglia in the dorsal horn of the spinal cord. This causes the upregulation of the P2X4 expression which is modulated by fibronectin and chemokine ligand 21 (CCL21). CCL2 signaling promotes P2X4 trafficking to cell surface of microglia. Influx of Ca^2+^ through ATP-stimulated P2X4 activates p38-MAPK and drives the synthesis and SNARE-dependent release of brain-derived neurotrophic factor (BDNF). After BDNF is released, it acts on its cognate receptor, trkB which consequently downregulates potassium-chloride cotransporter KCC2 expression in dorsal horn spinal lamina 1 neurons. This results in increase of intracellular [CI^-^] and leads to the reduction in anion gradient in dorsal horn which in turn induces depolarization of these neurons. The altered chloride gradient causes GABA to switch its effects from inhibition to excitation. The resultant hyperexcitability in the dorsal horn could underlie the increased sensitivity that is a feature of neuropathic pain.

P2X4 activation in spinal microglia leads to the release of brain-derived neurotrophic factor (BDNF), which communicates between microglia and spinal interneurons resulting in pain hypersensitivity via disinhibition of GABAergic input (Tsuda et al., 2003; Coull et al., 2005; Ulmann et al., 2008; Trang et al., 2009). Allodynia induced through peripheral nerve injury was reversed by pharmacological blockade of P2X4 receptors in the spinal cord (Tsuda et al., 2003) and P2X4^-/-^ mouse models have reduced inflammatory and neuropathic pain (Ulmann et al., 2008). These findings highlight the importance of P2X4 in microglial cells in the development of neuropathic pain, as reviewed by Inoue and Tsuda (2012) and Tsuda et al. (2013) and summarized in **Figure 1**. Furthermore, activated microglia play a critical role in the pathogenesis of other diseases such as spinal cord injury, stroke and neurodegenerative disease (i.e., Alzheimer’s and Parkinson’s disease), therefore P2X4 may also contribute to pathogenesis of these conditions.

Astrocytes are star-shaped glial cells providing a wide variety of functional support for neurons. P2X1-5 and P2X7 receptors have been identified at the transcriptional level *in situ* and *in vitro* in primary cultured rat cortical astrocytes, hippocampal astrocytes and astrocytes in the nucleus accumbens (Franke et al., 2001; Fumagalli et al., 2003; Dixon et al., 2004) and protein expression was further confirmed (Kukley et al., 2001). In rat hippocampus, immunostaining revealed expression of P2X4 particularly in S100β positive astrocytes in the CA1, CA3 and dentate gyrus regions (Kukley et al., 2001). However, in acutely isolated mouse cortical astrocytes no mRNA expression for P2X4 was detected using qRT-PCR. This correlated with electrophysiology studies showing insensitivity of ATP-induced currents to the positive modulator ivermectin (Jabs et al., 2007). At a functional level, P2X4 responses are not ubiquitously found since P2X4-mediated currents were not detected in acutely isolated cortical astrocytes (Lalo et al., 2008) or hippocampal slices (Jabs et al., 2007). Currently, therefore, there is limited evidence for P2X4 in astrocytes.

Oligodendrocytes are glial cells primarily responsible for myelination of neurons in the CNS and expression of several types of P2 receptor including P2X4 has been demonstrated in oligodendrocytes and in oligodendrocyte precursor cells (OPCs) (Agresti et al., 2005). Aside from neurons and neuroglial cells there is a vast network of blood vessels in the CNS. There is a long standing interest in the role of P2X4 in peripheral endothelial cells and the regulation of blood vessel tone (Yamamoto et al., 2000, 2006). P2X4 is abundantly expressed in peripheral vessel endothelial cells but it is unclear if this is also true for brain microvascular endothelial cells which play an important role in constitution of the blood brain barrier (BBB) and deterioration of this barrier is implicated in several neurological disorders. To date expression of P2X4 has been demonstrated in the hCMEC/D3 human microvascular endothelial cell line and the mouse microvascular cell line bend.3 (Bintig et al., 2012; Ozaki et al., 2016). Similar to the periphery, brain endothelial P2X4 may be involved in regulating shear stress responses in the cerebral vasculature and stimulation of protective factors such as osteopontin to enable ischaemic tolerance (Ozaki et al., 2016).

## Subcellular Localisation of P2X4

The cellular localisation of most P2X receptors is thought to be predominantly at the plasma membrane. However, P2X4 expression at the cell surface is limited with the majority of receptors occupying an intracellular residency in resting cells (Robinson and Murrell-Lagnado, 2013). P2X4 contains an additional C-terminal trafficking motif (YxxxGφ) which facilitates rapid internalization of cell surface P2X4 via AP2 and clathrin-dependent endocytosis (Royle et al., 2005). The subcellular distribution of P2X4 in many cell types shows a punctate, clustered staining pattern with overlapping markers of intracellular vesicles including recycling endosomes and lysosomes (Bobanovic et al., 2002; Qureshi et al., 2007; Stokes and Surprenant, 2009). Although regulated trafficking is not unique to P2X4 among the P2X receptor family, the ability of P2X4 to rapidly recycle between the plasma membrane and intracellular compartments is a robust feature studied by electrophysiological and biochemical methods. There is strong evidence that P2X4 receptors localize to the plasma membrane, endosomes, lysosomes, and vacuoles. Insights about dynamic P2X4 trafficking were revealed with use of a pH-sensitivecou fluorescent protein, pHluorin. Khakh and colleagues have recently used P2X4-pHluorin for *in vitro* trafficking studies to quantify and track P2X4 distribution within subcellular compartments (Xu et al., 2014). Similarly, other mechanistic studies also showed that P2X4 upregulation occurs both via altered trafficking and via increased P2X4 gene expression (Toulme et al., 2006, 2010; Raouf et al., 2007; Toulme and Khakh, 2012). Recently it was suggested that P2X4 functions as an ATP-gated channel in lysosomes (Huang et al., 2014) and regulates endolysosomal membrane fusion via Ca^2+^-dependent activation of calmodulin (Cao et al., 2015).

## P2X4 Downstream Signaling Pathways

Following ATP activation of P2X4 channels in the plasma membrane, the rapid influx of cations (Na^+^ and Ca^2+^) will cause membrane depolarisation. In addition the significant influx of Ca^2+^, as has been reported for P2X4, will likely trigger important downstream signaling pathways. For example, Ca^2+^ is important in neurons in the regulation of neurotransmitter release (Neher and Sakaba, 2008) and in various microglial functions (Farber and Kettenmann, 2006).

Recently, several lines of evidence suggest that P2X4 stimulated microglia signal to spinal lamina I dorsal horn neurons causing nociceptive output and that the critical microglia-neuron signaling molecule is BDNF (Coull et al., 2005). Notably, accumulation of BDNF in spinal dorsal horn microglia following nerve injury in global P2X4^-/-^ mice showed impaired ATP-evoked BDNF release which further demonstrated that P2X4 plays an important role in controlling BDNF release from microglia (Ulmann et al., 2008). The mechanistic gap between the activation of P2X4 and release of BDNF from microglia was bridged with a study from Salter and colleagues (Trang et al., 2009). Influx of Ca^2+^ through P2X4 in cultured microglia is a critical intracellular step linking stimulation of these receptors to the activation of p38 MAPK. This implicates p38 MAPK activation and BDNF release as a key step in microglia-neuron communication leading to nerve injury induced pain hypersensitivity. However, there may be other cell types expressing P2X4 involved in pain pathways. Peripheral macrophages also express P2X4 triggering Ca^2+^ influx and p38 MAPK phosphorylation (Ulmann et al., 2010). This signaling could further activate cytosolic PLA_2_ liberating arachidonic acid (AA) and release of prostaglandin E2 (PGE_2_). Consequently, the release of PGE_2_ leads to hypersensitivity of peripheral nociceptive pathways which is a hallmark of inflammatory pain (Basbaum et al., 2009).

## P2X4 in CNS Disorders

As knowledge regarding the expression and the characteristics of the individual P2X channels has emerged and expanded since they were first cloned in the late 1990s, questions have arisen over the importance of P2X channels in normal physiological functioning and their functioning in pathological conditions. Information regarding the specific role of P2X4 has been slower to accumulate largely due to the lack of selective antagonists for this P2X subtype (see P2X4 pharmacology section below). The first evidence for a pathological role for P2X4 was in pain processing. A landmark study by Tsuda and colleagues used antisense P2X4 oligonucleotides and described a major role for P2X4 in chronic pain and mechanical allodynia (Tsuda et al., 2003). The development of transgenic mice lacking P2X4 expression has since confirmed the role of this ion channel in modulation of pain signaling (Ulmann et al., 2008, 2010; Tsuda et al., 2009a).

## Chronic Pain

Chronic pain is described as persistent pain arising from a modification of pain processing pathways in the spinal cord and can be labeled as allodynia, hypersensitivity or neuropathic pain (Basbaum et al., 2009; Cohen and Mao, 2014). There are several review articles describing purinergic receptors in the context of pain and it is important to acknowledge here that more than one purinergic receptor has been implicated in pain – currently P2X2/3, P2X7 and P2Y1, P2Y2, P2Y12 are thought to be involved in the modulation of pain processing (Jarvis, 2010; Tsuda, 2016).

Tsuda and colleagues demonstrated that P2X4 was upregulated in spinal microglia after nerve injury (ligation of the fifth lumbar spinal nerve) and mediated tactile allodynia (defined as a hypersensitivity to non-painful stimuli) (Tsuda et al., 2003). Blocking P2X4 (with TNP-ATP) or knocking down P2X4 with antisense oligonucleotides reversed the allodynia phenotype and thus suggested that P2X4 may be a potential therapeutic target for this type of neuropathic pain. In 2005 it was demonstrated that communication between microglia and neurons was essential for the development of allodynia and that the critical factor involved was BDNF (Coull et al., 2005). Stimulation of spinal microglia with ATP induced BDNF secretion which through activation of TrkB receptors on spinal output neurons, alters E_anion_ causing disinhibition of GABAergic inputs from inhibitory spinal neurons. This leads to hyperexcitability of output neurons in the pain pathway. BDNF was already known to be a pro-nociceptive factor able to sensitize neurons (Kerr et al., 1999) but this study was the first to connect ATP-P2X4-BDNF to the neuronal shift in E_anion_. Ulmann et al. (2008) then provided direct evidence for P2X4 in mediating BDNF secretion from spinal microglia using a P2X4-deficient mouse generated by the Rassendren lab. They further demonstrated that P2X4^-/-^ mice did not develop mechanical hyperalgesia following peripheral nerve injury. This lack of mechanical hyperalgesia was confirmed using a second global P2X4 deficient mouse generated by the Ando lab. Behavioral tests demonstrated no difference in acute thermal, mechanical and chemical induced pain sensing in these P2X4^-/-^ mice but there was significant suppression of chronic inflammatory pain (induced by CFA injection) and allodynia due to nerve injury (Tsuda et al., 2009a).

More recently, P2X4 has been implicated in tolerance to morphine (Horvath et al., 2010) and morphine-induced hyperalgesia (Ferrini et al., 2013). Morphine enhanced microglial migration *in vitro* in a μ-opioid receptor dependent manner, and antisense P2X4 oligonucleotides reduced the development of morphine tolerance in rats (Horvath and DeLeo, 2009; Horvath et al., 2010). Furthermore, Ferrini et al. (2013) demonstrated morphine hyperalgesia required μ-opioid receptor-mediated upregulation of P2X4 in spinal microglia. Blocking the BDNF-TrkB pathway reversed the hyperalgesia. Therefore the microglial ATP-P2X4-BDNF axis seems to be a central pathway involved in the setting of pain thresholds. However, recent evidence has revealed that this mechanism may be restricted to males since female rodents did not display upregulation of P2X4 on microglia (Sorge et al., 2015; Mapplebeck et al., 2016).

## Alcohol-Related Disorders

In the search to identify therapeutic targets for Alcohol Use Disorders (AUD), several studies have demonstrated that P2X4 plays a role in alcohol-induced behavior (Yardley et al., 2012; Wyatt et al., 2014). P2X4 are expressed in key brain regions implicated in the reinforcing properties of alcohol and other drugs (Kimpel et al., 2007). This supports a role for P2X4 in alcohol addiction probably via modulation of P2X4 in the mesolimbic dopamine system, the brain reward system (Khoja et al., 2016). *In vitro*, P2X4 are inhibited by ethanol concentrations as low as 5mM and ethanol is thought to block the P2X4 channel in the open state but does not affect channel deactivation (Davies et al., 2005; Ostrovskaya et al., 2011). Crucial residues that mediates ethanol effects on P2X4 gating are Trp46, His241, Asp331 and Met336 (Xiong et al., 2005; Popova et al., 2010, 2013).

In P2X4^-/-^ mice heightened ethanol intake behavior is observed over wild-type counterparts suggesting that the P2X4 gene may be linked with alcohol intake and/or preference (Asatryan et al., 2011; Franklin et al., 2014). It has been shown that alcohol-preferring rats have lower expression of the P2X4 gene than alcohol-non-preferring rats, suggesting a correlation between P2X4 expression and alcohol intake (Kimpel et al., 2007; Franklin et al., 2015). Ivermectin, a broad-spectrum antiparasitic used worldwide in humans and animals, can antagonize the inhibitory action of ethanol on P2X4 and is suggested to interfere with ethanol binding to the channel (Asatryan et al., 2010). Other avermectins have been demonstrated to act in a similar fashion (Asatryan et al., 2014; Huynh et al., 2017). Studies also identify a role for P2X4 in regulating dopaminergic neurotransmission within the mesolimbic system. In dopaminergic neurons of the mesolimbic system, P2X4 has been shown to play a role in mediating alcohol-drinking behavior (Franklin et al., 2015). P2X4 receptor activity has also been linked to other behaviors of associated with dopaminergic neurotransmission, including motor control and sensorimotor gating (Khoja et al., 2016). Overall, these findings suggest that alcohol intake may be modulated by ethanol acting on P2X4 and that pharmacological potentiation of P2X4 by ivermectin or avermectin analogs may reduce alcohol consumption and preference.

## Neuroinflammatory Disorders

Neuroinflammation describes inflammatory changes in the brain and spinal cord associated with activation of the immune system (DiSabato et al., 2016). This is thought to be mediated by mediators such as chemokines, cytokines and second messengers (such as nitric oxide and prostaglandins). Resident immune cells such as microglia are involved, as are endothelial cells and astrocytes. Microglia regulate cytokines and inflammatory processes in the brain, typically elevated pro-inflammatory cytokines (IL-6, TNF-α, and IL-1β) and chemokines (CCL2, CCL5, CXCL1) define a state of neuroinflammation, as reviewed in DiSabato et al. (2016).

Neurodegenerative diseases such as Alzheimer’s disease and Parkinson’s disease are associated with neuroinflammation (Ransohoff, 2016) and microglia appear to be central (Saijo and Glass, 2011; Joers et al., 2016; Wes et al., 2016). P2X4 and the related purinergic channel P2X7 (Bhattacharya and Biber, 2016) are involved in the regulation of microglial pathways therefore may also play roles in exacerbating inflammation in the CNS. P2X4^-/-^ mice (Rassendren mouse model) have an attenuated inflammatory response associated with spinal cord injury with reduced NLRP1 inflammasome-related signaling pathways (de Rivero Vaccari et al., 2012). P2X4 deficiency also reduced the infiltration of peripheral immune cells (neutrophils and macrophages) to the spinal cord (de Rivero Vaccari et al., 2012).

P2X4 was up-regulated in microglial cells in an animal model of multiple sclerosis (autoimmune EAE in rats) (Vazquez-Villoldo et al., 2014). Using LPS as a model of neuroinflammation the P2X4 antagonists TNP-ATP and 5-BDBD were shown to reduce microglial activation *in vivo* and reduce the microglial loss in spinal cord. Following *i.c.v.* injection of LPS, the dentate gyrus region of the hippocampus showed classical hallmarks of microglial activation which were blocked by TNP-ATP (Vazquez-Villoldo et al., 2014).

Alcohol abuse is thought to enhance neuroinflammation (Potula et al., 2006) and the P2X4 receptor on microglial cells has been implicated (Gofman et al., 2014, 2016). In addition neuroinflammatory responses are also associated with ischaemic events in the brain and studies demonstrate upregulation of P2X4 in microglia following hypoxia and ischaemia (Wixey et al., 2009; Li et al., 2011). Recently, using a mouse model of middle cerebral artery occlusion (MCAO) endothelial P2X4 was required for the neuroprotective effect of ischaemic preconditioning (transient ischaemia followed by reperfusion) (Ozaki et al., 2016). P2X4 is known to be activated by shear stress generated by conditions of flow in peripheral endothelial cells (Yamamoto et al., 2000, 2006). Mice with a conditional knockout of vascular endothelial P2X4 showed severe deficits after MCAO due to an attenuation in release of the neuroprotective factor osteopontin (Ozaki et al., 2016).

## P2X4 Pharmacology

**FIGURE 2:**
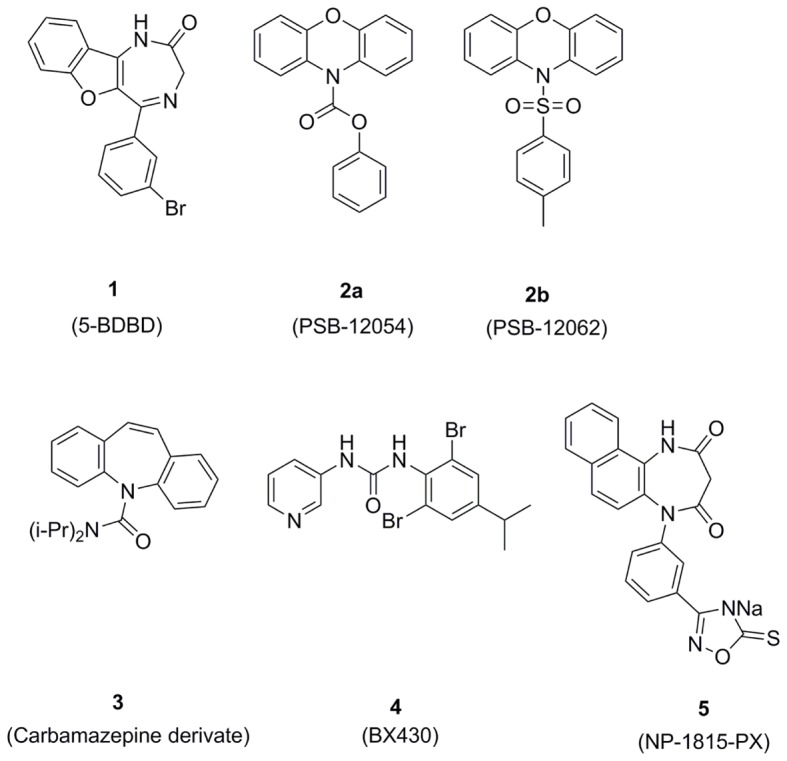
**Chemical structures of P2X4 antagonists**.

There has been significant progress in understanding P2X receptor pharmacology in recent years. Despite such advances, there remains a paucity of potent and selective pharmacological tools for some receptors. Agonists that selectively activate distinct members of this family are elusive, and with a few exceptions, progress has been slow when identifying selective inhibitors, some of which are shown in **Figure 2**. Here we describe what is currently known regarding pharmacological modulators of P2X4.

## Agonists

The most potent agonist of homomeric P2X4 is ATP. Dose-responses for ATP at rat and human P2X4 gave EC_50_ values of 6.9 ± 0.8 and 7.4 ± 0.5 μM, respectively (Soto et al., 1996a; Garcia-Guzman et al., 1997). In the original article characterizing rat P2X4 expressed in Xenopus oocytes the electrophysiological profile of agonists was ATP > 2-methylthio-ATP > CTP > α, β-methylene-ATP > dATP and no response was elicited by ADP, AMP, GTP, adenosine or the β,γ-methylene-ATP analog (Soto et al., 1996a). For human P2X4 expressed in Xenopus oocytes the same order of efficacy was demonstrated (Garcia-Guzman et al., 1997). BzATP is also known to act as a partial agonist at rat and human P2X4 (Bowler et al., 2003; Stokes et al., 2011).

## Positive Modulators

One of the most distinguishing features of P2X4 is potentiation by ivermectin – a macrocyclic lactone derived from the selective hydrogenation of avermectin B1, a natural product synthesized by *Streptomyces avermitilis*. Ivermectin is a mixture of 22,23-dihydroavermectin B1a and B1b in an approximate 80:20 ratio (Laing et al., 2017). Use of ivermectin as a successful anti-helminthic drug is due to its positive allosteric modulation of glutamate–gated chloride channels in nematode worms. Ivermectin also potentiates responses at vertebrate GABA(A) and nicotinic α7 receptors (Krůšek and Zemková, 1994; Krause et al., 1998). Of all purinergic receptors, P2X4 is the most sensitive to ivermectin (Khakh et al., 1999b) although a recent report claims some potentiation of human P2X7 responses by ivermectin (Nörenberg et al., 2012). Extracellular, but not intracellular, application of ivermectin (≤10 μM) potentiates P2X4 currents and delays channel deactivation (Khakh et al., 1999b; Priel and Silberberg, 2004). These observations are likely the result of ivermectin binding to separate sites on P2X4, a high affinity ivermectin binding site (pEC_50_ 6.6) which increases maximal current activated by saturating concentrations of ATP, and a low affinity site (pEC_50_ 5.7) which slows channel deactivation (Priel and Silberberg, 2004). Ivermectin appears to interact with the transmembrane domains of P2X4 and lateral fenestrations (Jelinkova et al., 2006; Silberberg et al., 2007; Hattori and Gouaux, 2012; Samways et al., 2012; Rokic et al., 2013) including a binding pocket which overlaps with an inhibitory ethanol binding site (Asatryan et al., 2010).

Avermectin analogs in addition to ivermectin also have positive modulator activity at P2X4. These include avermectin, emamectin, doramectin (Silberberg et al., 2007) and more recently abamectin, selamectin, and moxidectin (Asatryan et al., 2014; Huynh et al., 2017). Of these avermectin, abamectin and moxidectin have similar effects to ivermectin in preventing the action of ethanol on P2X4, but have less effect on GABA(A) receptors (Asatryan et al., 2014; Huynh et al., 2017).

There is also evidence for positive allosteric modulation of P2X4 by cibacron blue, an isomer of reactive blue-2, in functional studies. Potentiation of P2X4 responses were attributable to an increase in the apparent affinity of ATP for the rat P2X4 (Miller et al., 1998). This potentiation of P2X4 responses is smaller than that of ivermectin and was not demonstrated in cells expressing human P2X4 (Garcia-Guzman et al., 1997).

## Broad Spectrum Purinergic Receptor Antagonists

P2X4 receptors are generally less sensitive to the broad P2X antagonists, such as suramin and pyridoxalphosphate-6-azophenyl-2′,4′-disulfonic acid (PPADS). Suramin shows very weak activity at mouse, rat and human P2X4 orthologs with maximal inhibition of P2X4 currents ranging between 11 and 35% with pIC_50_ > 4 for all orthologs (Jones et al., 2000). In contrast, PPADS fully inhibits human and mouse P2X_4_ (pIC_50_ ∼ 5 for both), whilst the rat receptor is relatively insensitive to PPADS (Jones et al., 2000). It has been hypothesized that PPADS acts, in part, by forming a Schiff base with a lysine residue in P2X1 and P2X2. However, this residue in the P2X4 is replaced by a glutamate at the analogous position (Glu249) and when it is replaced by a lysine, the resultant P2X4 mutant is sensitive to inhibition by PPADS. Notably, the human P2X4 has only one lysine (Lys127), which is absent in the rat P2X4, conferring sensitivity to PPADS, and mutation of the residue to a lysine in the rat P2X4 (Asn127Lys) did not produce a PPADS-sensitive channel (Buell et al., 1996). Critically, the increased sensitivity of the human P2X4 to PPADS inhibition might not be only due to the different ability of PPADS to form Schiff base via lysine residues. In addition, several studies have confirmed that 2′,3′-O-(2,4,6-trinitrophenyl) ATP (TNP-ATP), which is a potent antagonist at P2X1, P2X3 and P2X2/3 receptors, acts as a weakly effective antagonist (IC_50_ 15.2 μM) at P2X4 receptors (Virginio et al., 1998). TNP-ATP is a competitive antagonist at P2X4 and can displace [^35^S]ATPγS binding to human P2X4 (Hernandez-Olmos et al., 2012; Abdelrahman et al., 2016). The potency of TNP-ATP at P2X4 estimated by Ca^2+^ influx assays in 1321N1 astrocytoma cells was 1.46–4.22 μM dependent on species (Abdelrahman et al., 2016).

## Divalent Cations and pH

With regard to ions, P2X4 is amongst the most sensitive P2 receptor to potentiation by zinc ions (Zn^2+^). Low concentrations (1–5 μM) of extracellular Zn^2+^ increases the gating efficiency of human or rat P2X4 without affecting the maximal response, however, high (>100 μM) extracellular Zn^2+^ inhibits P2X4 currents in a voltage-dependent fashion (Garcia-Guzman et al., 1997; Wildman et al., 1999; Acuna-Castillo et al., 2000). Similarly, the potentiating effects of Zn^2+^ are mimicked by Cd^2+^, however, Cu^2+^ modifies P2X4 activity in the opposite direction causing inhibition (Acuna-Castillo et al., 2000; Coddou et al., 2005). High concentrations of extracellular Cu^2+^ (300 μM) inhibit rat P2X4 in a non-competitive and voltage-independent fashion and this effect can be seen with other metal cations such as Hg^2+^ (Coddou et al., 2005). His140 plays a crucial role in the inhibitory action of Cu^2+^ but this was not required for the action of Zn^2+^ at P2X4 (Coddou et al., 2003). Moreover, Asp138 was further identified as important for Cu^2+^ binding at P2X4 and Cys132 was critical for Zn^2+^ potentiation at P2X4 (Coddou et al., 2007). These residues were validated by an NMR resolved structure of rat P2X4 extracellular domain as key residues that coordinate Cu^2+^ binding (Igawa et al., 2015).

The activity of P2X4 is not only regulated by metals and ions, but is also modulated by extracellular H^+^, such that pH < 6 completely inhibits channel activity and responses are potentiated above physiological pH (Wildman et al., 1999; Clarke et al., 2000). One key residue, His286, has been identified in conveying pH-sensitivity to P2X4 (Clarke et al., 2000). Inhibition by low pH may be an important physiological regulator of P2X4 receptor since P2X4 displays a predominant lysosomal distribution (Qureshi et al., 2007) where it appears to function as a lysosomal ion channel in addition to its plasma membrane role (Huang et al., 2014). In lysosomes the luminal pH is typically acidic with a resting pH 4.6 maintained by vacuolar H^+^-ATPases. Under these conditions P2X4 was not active but following alkalinisation of lysosomes with NH_4_Cl, ATP-induced responses could be recorded (Huang et al., 2014).

## Antidepressants

Recent studies have documented that some antidepressants may inhibit P2X4 receptors, however, this evidence remains controversial in view of contrasting findings. P2X4 is inhibited by several clinically used antidepressants including paroxetine, fluoxetine, desipramine, fluvoxamine, nortriptyline, and clomipramine (Nagata et al., 2009). Of these paroxetine is the most potent, non-competitively inhibiting human P2X4 heterologously expressed in 1321N1 cells at a pIC_50_ of 5.7 (Nagata et al., 2009; Abdelrahman et al., 2016). The studies suggest that such compounds may mediate analgesic effects in neuropathic pain models via the inhibition of P2X4 (Nagata et al., 2009; Zarei et al., 2014; Yamashita et al., 2016). However, paroxetine is much more potent as a serotonin reuptake inhibitor than an inhibitor of P2X4. There is also evidence that antidepressants may affect lysosomal trafficking of P2X4 in cerebellar microglial cells (Toulme et al., 2010). Furthermore, the hypothesis that the widely used tricyclic antidepressant amitriptyline may exert analgesic effects through P2X4 inhibition has also been tested (Sim and North, 2010). Amitriptyline applied acutely at 10 μM or by pre-incubation for several hours, had no effect on human P2X4 channel activity though modest inhibitory effects of 10 μM amitriptyline were observed at mouse and rat P2X4 (Sim and North, 2010). It is therefore highly unlikely that amitriptyline mediates analgesic effects via P2X4 inhibition in human subjects.

## Statins and Cholesterol Depleting Agents

Statins have been described as the most effective class of drugs to reduce serum cholesterol levels (Oesterle et al., 2017). The HMG-CoA reductase inhibitor fluvastatin inhibited the activity of heterologously expressed human P2X4 and native P2X4 in human monocytes (Li and Fountain, 2012). The activity of fluvastatin is likely to be due to depletion of cellular cholesterol as its inhibitory action was mimicked by methyl-β-cyclodextrin and filipin III (Li and Fountain, 2012). Some further studies have shown association of P2X4 within lipid rafts (Allsopp et al., 2010).

## Recent Advances in P2X4 Pharmacological Agents

Research surrounding P2X4 has been greatly hindered due to the lack of selective receptor antagonists. For a long time, researchers had to rely on non-selective P2X receptor antagonists such as TNP-ATP, BBG and paroxetine to study P2X4. However, these agents possess low affinity for P2X4 and are significantly more potent at other targets. Recently growing evidence that highlights the importance of P2X4 in health and disease has triggered interest in the development of selective receptor antagonists (**Table 1**). The discovery of selective P2X4 antagonists will, therefore, present a new avenue for scientific research to selectively target P2X4 therapeutically.

**Table 1:** P2X4 modulators and antagonists.

Compound	Nature	Potency at P2X4 IC_50_ μM (species)	Selectivity	Reference
5-BDBD	Competitive	1.6 (Human)	Not reported	Balazs et al., 2013; Abdelrahman et al., 2016
PSB-12054	Allosteric	0.189 (Human)	30-fold (P2X1)	Hernandez-Olmos et al., 2012
		2.10 (Rat)	50-fold (P2X2, 3, 7)	
		1.77 (Mouse)		
PSB-12062	Allosteric	1.38 (Human)	35-fold (P2X1-3, 7)	Hernandez-Olmos et al., 2012
		0.928 (Rat)		
		1.76 (Mouse)		
BX-430	Non-competitive allosteric	0.54 (Human)	10-fold (P2X1-3, 5)	Ase et al., 2015
			100-fold (P2X7)	
		1.89 (Zebrafish)		
Carbamazepine derivative	Negative allosteric modulator	3.44 (Human)	2-fold (P2X1, 3)	Tian et al., 2014
			30-fold (P2X2, 7)	
		54.6 (Rat)		
		14.9 (Mouse)		
NP-1815-PX	Not reported	0.26 (Human)	Not reported	Matsumura et al., 2016
Ivermectin	Positive allosteric modulator	0.25 (Human)	Acts on human P2X7	Priel and Silberberg, 2004; Nörenberg et al., 2012
Cibacron blue	Potentiator	Not determined	Not reported	Miller et al., 1998

## 5-BDBD

The benzodiazepine derivative 5-BDBD (5-(3-bromophenyl)-1,3-dihydro-2H-benzofuro[3,2-e]-1,4-diazepin-2-one) was one of the first selective P2X4 antagonists to be described in the literature (Balazs et al., 2013). The original experimental details are documented in a patent (Fischer et al., 2005), however, 5-BDBD is now commercially available. This compound has been reported as a moderately potent and selective P2X4 antagonist with an IC_50_ value of 1.2 μM in HEK-293 cells expressing human P2X4 (Balazs et al., 2013). The application of two different concentrations of 5-BDBD resulted in a rightward shift in ATP dose-response curve indicating that 5-BDBD acts as a competitive antagonist to P2X4 (Balazs et al., 2013). However, radioligand binding assays illustrated that 5-BDBD was unable to displace ^[35S]^ATPγS binding to P2X4 indicating that rather than acting as a true competitive antagonist, it has an allosteric mechanism at P2X4 (Abdelrahman et al., 2016).

## N-Substituted Phenoxazine Derivatives

More recently, compounds derived from N-substituted phenoxazine were identified as potent and selective allosteric P2X4 antagonists (Hernandez-Olmos et al., 2012). One of the derivatives, *N-*(Benzyloxycarbonyl) phenoxazine (PSB-12054), is an extremely potent antagonist of the human P2X4 (IC_50_ value of 0.189 μM), but is less potent toward rat P2X4 (IC_50_ value of 2.1 μM) and mouse P2X4 (IC_50_ value of 1.77 μM). It has been reported to possess high selectivity for human P2X4 with over 50-fold against P2X2, P2X3 and P2X7 and over 30-fold against P2X1 (Hernandez-Olmos et al., 2012). The second less potent, but more water-soluble, of the two derivatives is *N*-(p-methylphenylsulfonyl)phenoxazine (PSB-12062). PSB-12062 was shown to have similar potency in all three species: human P2X4 (IC_50_ value of 1.38 μM), rat P2X4 (IC_50_ value of 0.928 μM) and mouse P2X4 (IC_50_ value of 1.76 μM) (Hernandez-Olmos et al., 2012). PSB-12062 has been shown to be allosteric in nature with a 35-fold selectivity toward P2X4 versus P2X1, P2X2, P2X3, and P2X7. However, PSB-12062 was unable to completely block ATP-induced P2X4-mediated calcium influx even when used at high concentrations (>30 μM) (Hernandez-Olmos et al., 2012). A third compound also derived from N-substituted phenoxazine, PSB-12253, was used in a study on HUVECs to investigate P2X4 responses. The effects of this selective antagonist were confirmed by siRNA in HUVECs (Sathanoori et al., 2015).

## Carbamazepine Derivatives

Several carbamazepine derivatives were investigated as potential P2X4 antagonists, with N,N-diisopropyl-5H-dibenz[b,f]azepine-5-carboxamide being found to be the most potent toward human P2X4 (IC_50_ of 3.44 μM) although less potent at mouse and rat P2X4 (Tian et al., 2014). Despite its potency toward human P2X4, it appeared to lack selectivity against two other P2X subtype (P2X1 and P2X3) but was selective versus P2X2 and P2X7. Further optimization will be required to improve the selectivity of this compound.

## BX430

One of the most recently discovered P2X4 antagonists is the phenylurea BX430 (1-(2,6-dibromo-4-isopropyl-phenyl)-3-(3-pyriudyl)urea which has been shown through a patch-clamp study to have sub-micromolar potency (IC_50_ value of 0.54 μM) and a 10 – 100 fold selectivity toward P2X4 versus other P2X subtypes (Ase et al., 2015). Acting through a non-competitive allosteric route, BX430 has been shown to be able to potently antagonize zebrafish P2X4 but has no effect on rat and mouse P2X4 orthologs (Ase et al., 2015). BX430 was seen to completely abolish ivermectin facilitated pore formation and, according to the authors, BX430 is the only known compound to inhibit P2X4 mediated pore formation (Ase et al., 2015).

## NP-1815-PX

Screening a chemical library identified the compound NP-1815-PX (5-[3-(5-thioxo-4H-[1,2,4]oxadiazol-3-yl)phenyl]-1H-naphtho[1,2-b][1,4]diazepine-2,4(3H,5H)-dione) as a novel human P2X4 antagonist (IC_50_ value of 0.26 μM) (Matsumura et al., 2016). The inhibitory effect of NP-1815-PX was also shown on rat and mouse P2X4 and it showed selectivity against other P2X receptors (Matsumura et al., 2016). Intrathecal administration of NP-1815-PX alleviated nerve damage-associated mechanical allodynia in a chronic pain model and in a herpetic pain model without altering acute pain sensing (Matsumura et al., 2016).

## Future Perspectives

The discovery of new pharmacological tools, some of which are now available commercially, will facilitate our understanding of P2X4 cellular function in health and disease. In addition, such tools may provide chemical scaffolds for the development of new drugs that may be beneficial in the treatment of neuropathic pain. P2X4-selective antagonists with high potency, no species-restricted effect, water-solubility and, most importantly, an anti-allodynia effect in rodent chronic pain models remain to be identified.

## Author Contributions

LS initiated, designed, co-wrote, and edited the article. JL co-wrote the article and prepared the table. LB co-wrote and prepared the Figures. KD co-wrote the article. SF co-wrote and edited the article.

## Conflict of Interest Statement

The authors declare that the research was conducted in the absence of any commercial or financial relationships that could be construed as a potential conflict of interest.
